# GC-PROM: validation of a patient-reported outcomes measure for Chinese patients with gastric cancer

**DOI:** 10.1186/s12885-020-6518-z

**Published:** 2020-01-16

**Authors:** Xiaojuan Hu, Fen Zhao, Hongmei Yu, Yanhong Luo, Jinchun Liu, Yanbo Zhang

**Affiliations:** 1 Department of Health Statistics, School of Public Health, Shanxi Medical University, 56 South XinJian Road, Taiyuan, 030001 Shanxi Province China; 2 Department of Internal Medicine, Shanxi Provincial Peoples Hospital, 29 Twin Towers Temple Street, Taiyuan, 030012 Shanxi Province China; 3 Department of Internal Medicine, First Hospital of Shanxi Medical University, 85 South JieFang Road, Taiyuan, 030001 Shanxi Province China

**Keywords:** Gastric cancer, Patient-reported outcome, Classical test theory, Item response theory, Minimal clinically important difference

## Abstract

**Background:**

There is increasing recognition that PROs are important in the estimation of the burden of long-term survival among patients with gastric cancer. The study aimed to develop a disease-specific instrument to assess patient-reported outcomes for Chinese patients with gastric cancer.

**Method:**

Following the FDA’s draft guidance for patient-reported outcome, conceptual framework and item pool were defined based on relevant existing work. A draft scale was formed after revising some items based on feedback from experts and Chinese patients with gastric cancer. The pre-survey and formal survey were conducted in eight different hospitals in Shanxi Province, and two item-selection process based on classical test theory and item response theory. Finally, the patient-reported outcomes measure for Chinese patients with gastric cancer (GC-PROM) was validated in terms of reliability, validity, and feasibility. The minimal clinically important difference was determined by distribution-based method.

**Results:**

The final GC-PROM consisted of 38 items, 13 subdomains, and 4 domains. Reliability was verified by Cronbach’s alpha coefficient for four domains and 13 subdomains respectively. The validity results showed that the multidimensional scale fulfilled expectations. In the formal survey, the completion rate was 96.16%, and the average filling time was less than half an hour. The values of the minimal clinically important difference were 4.14, 3.41, 3.37, and 3.28 in the four domains.

**Conclusions:**

The GC-PROM had good reliability, validity, and feasibility and thus can be considered an effective clinical evaluation instrument for Chinese patients with gastric cancer.

## Background

Gastric cancer (gastric carcinoma, GC) is a malignant tumor occurring in the epithelial tissue of the stomach. GC accounts for more than 95% of malignant tumors of the stomach [1]. There are approximately 989,000 new patients with GC worldwide each year, but the incidence of the disease varies greatly by region [2]. Although the diagnosis and treatment of GC are developing, the 5-year survival rate for patients with GC is only 20%. In China, GC is a major public health problem [3]. GC causes physical pain to patients, poor mental state, and enormous costs for many families, which reduce the Chinese patients’ quality of life (QoL). So many patients with GC are focusing more on how improving overall QoL [4].

In recent years, patients’ subjective feelings about treatment have been an important part of the improving patients’ QoL [5]. However, earlier methods were unable to measure patients’ self-reported results, such as physician report [6]. Therefore, new patient-generated reports, also known as patient-reported outcomes (PROs), are now used to assess the overall burden of cancer and the effectiveness of interventions. PROs involve reports taken directly from patients regarding their health status, functional status, and treatment experience [7]. In medical care for patients with GC, functional effects have usually been separated into three categories: physiological, psychological, and social. It is possible that treatments may also cause physical discomfort to patients, testing the psychological endurance of both patients and their families [8]. Economic effects have sometimes also been discussed in the functional effects of illness [9]. To select the best therapeutic schedule, it is necessary to carry out a comprehensive assessment of various plans.

At present, the main disease-specific instruments of GC that have been developed are the EORTC quality of life questionnaire-stomach cancer (EORTC QLQ-STO52) [10], the Functional Assessment of Cancer Therapy-gastric (FACT-Ga) [11], quality of life instruments for cancer patients-stomach cancer (QLICP-ST) [12], and the Special Symptom Scale developed by Chen-wun in Taiwan, China [13]. EORTC QLQ-STO 52, FACT-Ga, and QLICP-ST was developed by combining general module with special module. The Chinese version of EORTC QLQ-STO52 and FACT-Ga had been culturally debugged and evaluated [14]. But there were still some items that might not suitable for Chinese culture. QLICP-ST was a gastric cancer scale developed for Chinese cancer patients. However, the disease-specific items might be less than those in the EORTC QLQ-STO52. It had few specific items on the effectiveness, compliance, satisfaction, and side effects in the field of cancer treatment [15]. The Special Symptom Scale developed by Chen-wun also didn’t divide domains [13].

In sum, there are already many reliable scales for measuring the QoL of patients with GC worldwide. However, if used alone, these scales are often not specific enough and cannot be roundly used to measure the QoL of Chinese patients with GC [16]. Additionally, because of QoL strongly dependent on cultural background, foreign scales cannot be used directly after translation. Because of economic and cultural differences across regions of China, Chinese-developed instruments for patients with GC have not been widely used [17]. Therefore, it was necessary to develop the PROM for Chinese patients with GC to focus more on the related aspects of the treatment as it is perceived by patients. In addition to laboratory and imaging methods, the data from PROM can be used to improve the reliability of clinical efficacy evaluations by comprehensively measuring many aspects of patient-reported health [18]. As a result, PROs are able to provide a reference for doctors in their diagnosis and treatment practices [19]. Prior to using PRO measures in clinical practice and research, the instruments need to be cautiously developed and validated to avoid biased results that might lead to incorrect interpretations [20].

## Methods

### Setting

The two surveys (i.e., pre-survey and formal survey) were carried out in eight hospitals in Shanxi Province, China. These hospitals were the First Hospital of Shanxi Medical University, the Second Hospital of Shanxi Medical University, Shanxi Cancer Hospital, the 264 Hospital of Chinese People’s Liberation Army (PLA), the 17th Hospital of the Chinese Railway, the People’s Hospital of Gaoping City, the People’s Hospital of Zezhou City, and the Fourth People’s Hospital of Linfen City.

### Sample

Before collecting samples, investigators contacted related departments of target hospitals and communities to get support from hospital staff and community workers. Preparations were also made to publicize the study through posters in hospital departments and communities. The documents introducing the survey were distributed. From July 2015 to September 2015, patients diagnosed with GC were recruited. The inclusion criteria for patients with GC were as follows: patients who had been diagnosed with GC, were over 18 years old. The exclusion criteria were as follows: patients with other serious disease; patients with disturbance of consciousness; patients who were unable to understand to complete the questionnaire for any reason. We simultaneously selected healthy subjects who lived in the same communities as the patients. Healthy subjects met the following criteria: They were not suffering from other diseases of the digestive system, other malignant tumors, or mental illness; were similar in age to the patients with GC; and they volunteered to participate in the investigation.

### Development and formation of GC-PROM

**Fig. 1:**
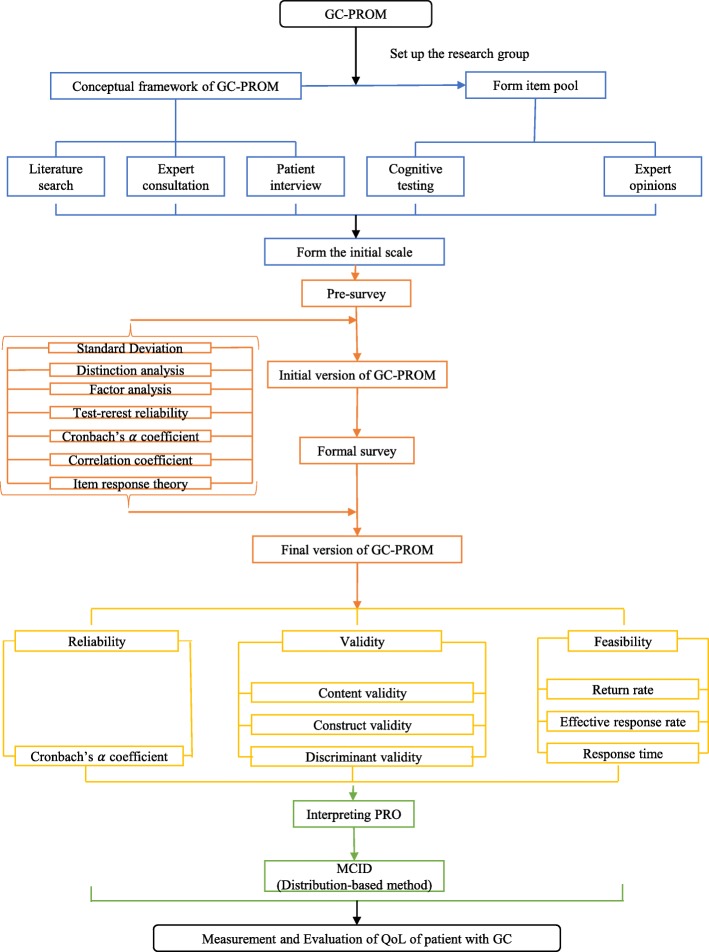
A flowchart of three-phase developmental process

The GC-PROM was developed in three phases [21], and details of each phase are described below. Figure 1 presented a flowchart of three-phase development process.

### Phase 1: identification of conceptual framework and items

#### Literature searches and patient interviews

Literature searches were carried out on network databases for keywords such as PRO measure, PRO scale, PRO instruments, and gastric cancer. Using the principles of FDA on the PROM and search results, we established a conceptual framework for GC-PROM including four domains and 13 subdomains. We conducted face-to-face interviews with 10 patients with GC. Researchers wrote down the interviewees’ original words as far as possible. After the interview, all information was sorted and an initial pool was developed.

#### Cognitive test and expert consultation

Other 10 hospitalized patients with GC took part in a cognitive test of the questionnaire. The group included seven men and three women, with an average age of 54 years. We also sought views from experts. In the final step, we integrated the views of experts and patients to modify the items and develop the draft version of GC-PROM.

#### Scale scoring

The response options of items used five-point Likert scoring scales, with scores ranging from zero to four points, including positive items (items with higher QoL) and negative items (items with lower QoL). For the convenience of calculation, positive items were recoded as the original score plus one point. The negative items were recoded as five minus the original score [22]. The higher total scores of the subdomain, the better the patients’ QoL.

### Phase 2: formation of initial and final scales using two item-selection processes

During the formation process of GC-PROM, seven methods were used to select items through two item-selection processes. The first six methods were based on classical test theory (CTT). The IRT was used as the seventh method. One of IRT models (i.e., Samejima’s Graded Response Model) were the preferred methodology for statistically analyzing patients’ latent traits [23]. An item was considered for selection if it was retained by six or more methods. An item’s practical significance was considered before deleting in the pre-survey. If it was meaningful in fact, the item would be temporarily retained and screened in the formal survey. We finally removed this item when it was still suggested to be deleted.

#### Statistical methods

Seven methods were used to evaluate the items:

1. When the standard deviation (SD) of an item was ≤1, the corresponding item was deleted [24].
2. We deleted items with factor loading that were low (< 0.4) or close to other factors in the exploratory factor analysis [25].
3. An item was considered for deletion when the Pearson correlation coefficient for the item and its subdomain was < 0.60 or the Pearson correlation coefficient for the item and another subdomain was > 0.50 [25].
4. An item was considered for deletion when the corrected item-total correlation was < 0.50 and the item’s deletion increased the value of Cronbach’s alpha coefficient [24].
5. Items with smaller correlation coefficients of retest reliability (< 0.6) were removed [26].
6. Each item score of patients and healthy subjects was analyzed using a *t*-test to distinguish the items in distinction analysis. Deletion was recommended for items with *P* values > 0.05 [23].
7. In the Graded Response Model, the practical values of the item parameters for deletion were as follows: item discrimination parameter (a) < 0.4 or difficulty parameter (b) ∉ (− 3, 3) [27].

### Phase 3: evaluation of measurement properties

The properties of the final GC-PROM version were assessed by using data from a formal investigation.

#### Evaluation of reliability

The internal consistency of the GC-PROM was assessed by using Cronbach’s alpha coefficients of 13 subdomains. Generally, a value of more than 0.70 indicated that it had a good internal consistency [28].

#### Evaluation of validity

##### Content validity

The relevant literature, subjects’ opinions, and experts were consulted in establishing the content validity, which represents how well the items captured the concept of interest [29].

##### Construct validity

Confirmatory factor analysis was used to examine the structure of the GC-PROM. The standardized factor loadings for an item should be greater than 0.5 [30].

##### Discriminant validity

Discriminant validity is the ability of an instrument to measure a difference between two groups. The *t*-test was used to compare differences between patients with GC and healthy subjects, with the significance level set at *P* < 0.05 [31].

#### Evaluation of feasibility

Feasibility mainly reflects the acceptability of the GC-PROM. The return and response rate of the questionnaires was rationalized with the general requirement set at ≥85%. The questionnaire completion time was generally less than half an hour. We also took the proportion of miss data and maximum endorsement frequencies [32].

### Interpretation of PRO results: minimal clinical important difference (MCID)

MCID was designed to solve the clinical explanation problem of a GC-PROM score change [33]. The methods used to estimate the MCID mainly include the effect size (ES), standard error of measurement (SEM), standardized response mean, and reliable change index (RCI) [34]. In this article, we used SEM and RCI to estimate the MCID.

## Results

### Participant characteristics

A total of 145 patients and 55 healthy subjects were included in the pre-survey. Among these subjects, 20 patients completed the questionnaire again 4 days after first completing the questionnaire. Finally, completed questionnaires were collected from 130 patients and 52 healthy subjects. All 20 retest questionnaires were recovered. In the formal survey, a total of 530 questionnaires (400 patients with GC, 130 healthy subjects) were administered. Ultimately, completed questionnaires were collected from 364 patients with GC and 112 healthy subjects. A total of 45 patients with GC were retested, and all of the retest questionnaires were recovered. We compared baseline data of two groups using *t*-tests for continuous variables and chi-square tests for categorical variables. The results with the significance level set at *P* < 0.05 showed that the baseline data from patients with GC and from healthy subjects were all comparable (Table 1).

**Table 1:** Baseline data of subjects in the formal survey

Variables	Group	Case	Control	t / *x*^2^	*P*
Home-places	City	109	39	0.951	0.330
	countryside	255	73		
Age (X ± *s*)		57.14 ± 10.08	55.78 ± 9.55	1.265	0.207
Gender	Female	266	74	2.060	0.151
	Male	98	38		
Height (cm, X ± *s*)		164.51 ± 9.21	164.54 ± 7.52	−0.032	0.974
Weight (kg, X ± *s*)		57.37 ± 11.14	59.17 ± 9.15	−1.557	0.120
Marital status	Single	13	9	5.321	0.256
	Married	297	82		
	Separated	19	7		
	Divorced	7	3		
	Widowed	28	11		
Occupation	Peasant	174	45	2.395	0.880
	Worker	67	26		
	Clerk	33	11		
	Professionals	22	7		
	Management	19	6		
	self-employed	13	5		
	Other	36	12		
Monthly income	<$150	196	55	0.951	0.813
	$150~$450	119	39		
	$450~$750	34	13		
	>$750	15	5		

### The conceptual framework of the GC-PROM

The established conceptual framework included four domains, 13 subdomains. After the literature review and interviews with patients with GC, an initial pool of 79 items was developed. Based on the cognitive test and expert consultation, we deleted 14 items, added three items, and modified two items. Finally, conceptual framework included the scale contained 4 domains (physiological, psychological, social, and therapeutic domains), 13 subdomains (abdominal symptoms, systemic symptoms, physical state, independence, anxiety, depression, pessimism, fear, social support, social adaptation, effectiveness, satisfaction, compliance, and drug side effects), and 68 items.

### Formation of the initial and final scales through two item-selection processes

Seven methods, including the SD, exploratory factor analysis, Cronbach’s alpha coefficient, retest reliability, correlation coefficient, distinction analysis, and IRT, were used to select items. Twenty-two items in the selected item pool were suggested for deletion by seven methods. Meanwhile practical meanings of 22 items were taken in account. Finally, a consensus was reached that these items should be deleted. In the second item-selection process, a formal investigation was conducted with the above reduced (i.e., 46 items) questionnaire. The items were again screened using the above seven methods and practical meanings. According to the results shown in Table 2, eight items were deleted.

**Table 2:** Screening results of the second item-selection phase using CTT and IRT

Item	IRT					SD	Factor analysis	CITC	Retest reliability	Correlation coefficient	*P*	Outcome
	α	b_1_	b_2_	b_3_	b_4_							
PHD1	3.23	−1.67	−0.59	0.24	1.25	1.141	**0.342**	0.587	0.923	0.702	0.001	√
PHD2	3.51	−1.78	−0.70	0.07	0.98	1.179	**0.351**	0.601	0.887	0.716	0.001	√
PHD3	0.80	−3.63	−1.48	0.71	**3.08**	1.060	0.749	**0.454**	0.882	**0.585**	0.001	×
PHD4	0.96	**−3.94**	−2.47	−0.78	1.14	1.063	**0.082**	**0.463**	0.915	**0.593**	0.001	×
PHD5	1.33	**−3.53**	−2.92	−1.71	−0.19	**0.923**	**0.191**	0.525	0.791	0.629	0.001	×
PHD6	1.13	−2.99	−1.25	0.02	2.21	1.066	0.684	0.577	0.839	0.687	0.001	√
PHD7	0.83	−2.81	−1.32	0.33	2.67	1.181	0.610	**0.489**	0.927	0.628	0.001	√
PHD8	1.19	**−3.62**	−2.49	−1.06	0.77	1.001	0.571	0.588	0.882	0.690	0.001	√
PHD9	0.42	**−7.06**	−4.04	− 0.50	**3.28**	1.093	0.443	**0.325**	0.928	**0.479**	0.001	×
PHD10	3.25	**−3.10**	−2.39	−1.35	−0.09	**0.758**	0.714	0.599	0.818	0.817	0.001	√
PHD11	2.00	−2.72	−2.01	−1.15	0.39	**0.937**	0.571	0.500	0.795	0.814	0.001	√
PHD12	1.29	**−4.66**	−3.32	−1.85	−0.05	**0.798**	0.467	**0.419**	0.805	0.725	0.001	×
PHD13	1.26	−2.28	−1.06	0.57	2.09	1.103	0.511	**0.496**	0.727	0.769	0.001	√
PHD14	9.30	−1.60	−0.60	0.38	1.39	1.019	0.437	0.772	0.846	0.901	0.001	√
PHD15	3.46	−1.69	−0.81	0.10	1.22	1.102	**0.330**	0.700	0.826	0.876	0.001	√
PHD16	5.25	−0.92	−0.14	0.79	1.73	1.129	0.814	0.799	0.905	0.946	0.001	√
PHD17	4.32	−1.58	−0.50	0.31	1.11	1.176	0.780	0.799	0.955	0.951	0.001	√
PSD1	3.32	−1.49	−0.37	0.53	1.48	1.124	0.830	0.691	0.809	0.875	0.001	√
PSD2	3.77	−1.02	−0.11	0.78	1.49	1.195	0.869	0.713	0.844	0.894	0.003	√
PSD3	1.18	**−4.14**	−1.94	−0.44	1.66	**0.946**	0.456	**0.465**	0.866	0.715	0.001	×
PSD4	2.41	−2.48	−1.49	−0.32	0.64	1.031	0.788	0.683	0.866	0.805	0.001	√
PSD5	3.33	−1.87	−1.04	−0.11	0.85	1.091	0.799	0.757	0.914	0.858	0.002	√
PSD6	3.41	−1.79	− 1.07	−0.19	0.86	1.101	0.757	0.744	0.921	**0.851**	0.001	√
PSD7	2.88	−2.68	−1.47	−0.57	0.81	**0.917**	0.754	0.718	0.865	**0.817**	0.005	×
PSD8	1.21	**−3.09**	−1.68	−0.14	1.81	1.022	0.544	**0.485**	0.886	0.663	0.001	√
PSD9	2.90	−2.30	− 1.33	−0.38	0.50	1.085	0.832	0.755	0.834	0.887	0.001	√
PSD10	3.52	−1.84	−0.86	0.01	0.93	1.149	0.849	0.781	0.822	0.906	0.001	√
PSD11	5.12	−1.80	−1.01	−0.30	0.59	1.145	0.835	0.814	0.743	0.921	0.001	√
SOD1	4.27	**−5.58**	−1.78	−1.13	−0.05	**0.827**	0.883	0.740	0.860	0.868	0.006	√
SOD2	5.00	−2.14	−1.24	−0.62	0.34	1.044	0.908	0.761	0.905	0.905	0.001	√
SOD3	1.86	−2.02	−0.99	0.30	1.50	1.079	0.813	0.622	0.835	0.844	0.001	√
SOD4	1.01	**−4.17**	−2.97	−1.86	0.18	**0.948**	0.522	**0.399**	0.881	**0.575**	0.001	×
SOD5	1.41	−2.30	−0.97	0.39	1.80	1.117	0.717	0.569	0.838	**0.730**	0.001	√
SOD6	6.85	−1.53	−0.68	0.14	1.13	1.126	0.926	0.842	0.737	**0.909**	0.001	√
SOD7	5.99	−1.66	−0.72	0.21	1.07	1.111	0.918	0.834	0.711	0.904	0.001	√
SOD8	2.52	−1.59	−0.89	0.08	1.15	1.176	0.813	0.662	0.764	0.801	0.001	√
THD1	3.63	−1.99	−1.19	0.00	1.11	**0.983**	0.868	0.780	0.804	0.898	0.001	√
THD2	7.16	−1.76	−0.78	0.24	1.12	1.031	0.863	0.829	0.776	0.926	0.001	√
THD3	2.78	−2.06	−0.93	0.12	1.00	1.098	0.780	0.746	0.696	0.893	0.001	√
THD4	2.04	−**3.18**	− 2.14	−0.22	1.20	**0.854**	0.657	0.544	0.880	0.885	0.001	√
THD5	2.85	−2.81	−1.62	0.21	1.50	**0.812**	0.601	0.544	0.736	0.872	0.001	√
THD6	3.61	−2.65	−1.64	−0.79	0.48	**0.888**	0.868	0.780	0.811	0.908	0.001	√
THD7	13.24	**−4.71**	−2.08	−0.76	0.24	**0.782**	0.936	0.893	0.826	0.951	0.001	√
THD8	5.49	**−4.47**	−1.89	−0.71	0.35	**0.826**	0.898	0.833	0.901	0.926	0.006	√
THD9	2.76	−2.14	−1.32	0.21	1.29	**0.967**	0.860	0.574	0.850	0.880	0.001	√
THD10	2.19	−1.81	−0.82	0.51	1.96	1.027	0.860	0.574	0.918	0.894	0.001	√

“√"was represented the selected item. “×” represented the item considered to be deleted. Bold word indicated values did not meet the criteria

*PHD* physiological domain, *PSD* psychological domain, *SOD* social domains, *THD* therapeutic domain, *IRT* item response theory, *SD* standard deviation, *CITC* corrected item-total correlation

Finally, the scale contained 4 domains, 13 subdomains, and 38 items (See Additional file 1). The structural framework of the final scale was shown in Table 3.

**Table 3:** Scale structure of the final GC-PROM

Domains	Subdomains	Item
Physical domain	Abdominal symptoms	1-, 2-, 3-, 4-, 5-
	Systemic symptoms	6-, 7-
	Physical state	8-, 9-, 10-
	Independence	11+,12+
Psychological domain	Anxiety	1-, 2-
	Depressed	3-, 4-, 5-,6-
	Pessimism	7-, 8-, 9-
Social domain	Social support	1+, 2+, 3+
	Social adaptation	4+, 5+, 6+, 7+
Therapeutic domain	Effectiveness	1+, 2+, 3+
	Satisfaction	4+, 5+
	Compliance	6+, 7+, 8+
	Drug side effects	9-, 10-

Negative items were denoted by “-”. Positive items were denoted by “+”

### Evaluating the properties of the GC-PROM

The final GC-PROM was evaluated for validity, reliability, and feasibility using data obtained from 364 patients with GC and 112 healthy subjects.

#### Evaluation of reliability

Cronbach’s alpha coefficients for the four domains and 13 subdomains were between 0.700 and 0.917. As was evident in these values, the GC-PROM demonstrated a good degree of internal consistency reliability.

#### Evaluation of validity

##### Content validity

To ensure that all the items appropriate, we assessed content validity by referring to the relevant previous literature. Face-to-face interviews were conducted with patients with GC to identify potential items. Meanwhile, we also consulted with experts for item refinement.

##### Construct validity

The indexes of fit for four domains (Root Mean Square Residual: 0.048–0.079; Normed Fit Index: 0.91–0.97; Bentler Comparative Fit Index: 0.91–0.98, incremental fit index: 0.91–0.98.) met the defined criteria, which were strongly suggested by the high factor loading. The results of confirmatory factor analysis appear in Table 4. The standardized factor loadings of 13 subdomains were greater than 0.5. Therefore, the construct validity was deemed satisfactory.

**Table 4:** Results of the CFA

Subdomains	Item	Nonstandard Factor Loading	Standard Factor Loading	standard error	*t*
Abdominal symptoms	PHD1	1.00	0.87	0.05	19.57
	PHD2	1.01	0.86	0.05	19.04
	PHD3	0.61	0.50	0.06	9.35
	PHD4	0.60	0.50	0.06	7.77
	PHD5	0.50	0.50	0.05	9.70
Systemic symptoms	PHD6	0.56	0.74	0.04	13.06
	PHD7	0.68	0.72	0.05	12.75
Physical state	PHD8	0.63	0.57	0.06	11.40
	PHD9	0.92	0.91	0.04	20.671
	PHD10	0.95	0.86	0.05	19.26
Independence	PHD11	1.04	0.92	0.05	20.13
	PHD12	1.02	0.87	0.05	18.51
Anxiety	PSD1	0.96	0.85	0.05	17.68
	PSD2	1.02	0.86	0.06	17.79
Depressed	PSD3	0.78	0.75	0.05	16.04
	PSD4	0.92	0.84	0.05	18.86
	PSD5	089	0.81	0.05	17.78
	PSD6	0.54	0.51	0.05	10.23
Pessimism	PSD7	0.88	0.81	0.05	18.18
	PSD8	0.97	0.84	0.05	19.17
	PSD15	1.03	0.90	0.05	21.27
Social support	SOD1	0.70	0.85	0.04	18.21
	SOD2	0.94	0.90	0.05	19.64
	SOD3	0.72	0.66	0.05	13.54
Social adaptation	SOD4	0.68	0.61	0.05	12.43
	SOD5	1.05	0.94	0.05	22.91
	SOD6	1.02	0.92	0.05	22.26
	SOD7	0.89	0.76	0.05	16.74
Effectiveness	THD1	0.83	0.85	0.04	19.39
	THD2	0.93	0.90	0.04	21.18
	THD3	0.91	0.83	0.05	18.61
Satisfaction	THD4	0.72	0.84	0.05	15.16
	THD5	0.53	0.65	0.04	11.97
Compliance	THD6	0.73	0.82	0.04	18.70
	THD7	0.75	0.96	0.03	24.32
	THD8	0.74	0.90	0.03	21.60
Drug side effects	THD9	0.77	0.79	0.06	12.27
	THD10	0.74	0.72	0.06	11.52

PHD: physiological domain. PSD: psychological domain. SOD: social domains. THD: therapeutic domain

##### Discriminant validity

The results of discriminant validity are shown in Table 5. The results of discriminant validity (*P* values < 0.05) suggested that the GC-PROM was an appropriate instrument to distinguish between patients and healthy subjects.

**Table 5:** Scores comparisons between healthy subjects and patients with GC (X ± *s*)

Subdomains	Patients with GC	healthy subjects	Cohen’s d	t/ *t*^,^	*P*
Abdominal symptoms	17.09 ± 4.06	23.41 ± 1.33	2.09	16.210	< 0.001
Systemic symptoms	8.48 ± 1.49	9.37 ± 0.83	0.73	6.077	< 0.001
Physical state	9.50 ± 2.73	14.24 ± 1.05	2.29	17.942	< 0.001
Independence	5.82 ± 2.19	9.04 ± 1.24	1.81	14.875	< 0.001
Anxiety	5.65 ± 2.16	9.46 ± 0.92	2.30	18.189	< 0.001
Depressed	14.39 ± 3.42	19.39 ± 0.65	2.03	15.371	< 0.001
Pessimism	10.97 ± 3.06	14.94 ± 0.31	1.83	13.692	< 0.001
Social support	11.57 ± 2.57	14.03 ± 1.18	1.23	9.778	< 0.001
Social adaptation	13.24 ± 3.86	19.29 ± 0.92	2.16	16.424	< 0.001
Effectiveness	10.26 ± 2.82	13.87 ± 1.20	1.67	13.178	< 0.001
Satisfaction	7.12 ± 1.46	8.20 ± 1.06	0.85	7.228	< 0.001
Compliance	12.34 ± 2.31	13.76 ± 1.42	0.74	6.162	< 0.001
Drug side effects	6.46 ± 1.77	9.15 ± 0.80	1.96	15.636	< 0.001

#### Evaluation of feasibility

In this formal survey, the return and response rate of questionnaires were 93.40 and 96.16%, respectively. The average completing time was less than half an hour. No major floor or ceiling effects were found. The maximum proportion of participants who endorsed a single category for each item was less than 80%. Only 3.84% of the responses to individual items were missing. We tested the missing questionnaire data using Little’s Missing Completely at Random Test. The test showed that the data were missing at random, and we filled them in using the Expectation-Maximization Algorithm.

### MCID

From statistical results of Table MCID, the value of the MCID was greater when determined using the RCI than when it was determined using the SEM. Therefore, the value of MCID determined using the RCI was chosen as the final judgment. We finally identified the minimum clinical values of 4.14, 3.41, 3.37, and 3.28 in the physiological, psychological, social, and therapeutic domains, respectively.

## Discussion

There is increasing recognition that PROs are important in the estimation of the burden of long-term survival among patients with GC. In this environment, it is essential to get more acquainted with information regarding patients’ QoL [3]. Therefore, the present study developed a reliable and valid patient-reported scale for patients with GC in China. Using the currently available PRO instruments as a starting point, we developed the GC-PROM to assess the QoL of patients with GC. The GC-PROM comprises four domains, 13 subdomains, and 38 items. The results of our study indicated that the GC-PROM is a valid instrument for measuring quality of life among patients with GC. The application of PROs in the evaluation of curative effects could make clinicians more aware of the patient’s situation and provide a reference for diagnosis and treatment [7].

Quality of life research conducted in China has historically involved the use of questionnaires that have been translated from another language. As a result some of the items have been inconsistent with some habits typical of Chinese people; particularly habits pertaining to inherently personal practices, or questions about habits that many Chinese people would consider to be sensitive areas of inquiry—resulting in potential bias [17]. The scale developed in the current study via discussion with specialists and interviews with patients with GC addresses this applicability problem with regard to patients in China. The GC-PROM is characterized by taking the therapeutic field and family relationships as independent domains, in contrast to other GC questionnaires. The measurement of satisfaction with treatment that patients received is the main focus in new drug clinical trials [9]. These subdomains (i.e., effectiveness, compliance, drug side effects) can provide related information about the effects of the targeted drug on patients’ quality of life and identify the acceptance of new drug among patients. Researchers can promote clinical therapeutic drug development and select an optimal therapy based on information and data gained. In the social field, family relationship is emphasized to recognize the importance of family support during the recovery of patients.

Exploratory factor analysis was carried out in the four domains based on one-dimensional assumption of the IRT [27]. The Kaiser-Meyer-Olkin values in four domains were 0.822, 0.875, 0.761, and 0.774 in the first item-selection process. The *P* value of Bartlett’s spherical test was < 0.001, indicating that the data were suitable for factor analysis. Four factors, three factors, two factors, and four factors with characteristic root greater than 1 were extracted from physical, psychological, social and therapeutic domains respectively. The factor analysis also showed that each factor (i.e., subdomain) had the unidimensionality. The method of GRM ran on the items of each subdomain.

There were many methods used in the selecting items. A variety of methods were used to ensure the quality of the selection and to make selected items more representative, independent, and sensitive. Previous research mostly used the method of CTT for item selection. Recently, IRT has gradually gained popularity for selecting items [23]. GRM is one of the most commonly used IRT models, and is suitable for Likert-type scales. The GRM method was used as a criterion for selecting items in our study. The significance of IRT is that it can guide item selection and test construction. The information function of IRT can be used to describe items’ measurement validity, which can be used as direction for the formation and modification of these items [24]. Therefore, the present study used IRT in the process of creating the GC-PROM.

To obtain reliable and accurate parameter estimates, some scholars have suggested that the sample size should be 5 to 10 times the number of observed variables in a factor analysis [20]. Most previous work that has applied item response theory (IRT) has not specified the sample size [35]. We conducted a pre-survey among a small sample (145 patients with GC and 55 healthy subjects) using a 68-item questionnaire. The purpose of this pre-survey was to ask patients how they felt about the GC-PROM items. This avoided ambiguity in understanding and reduced omission of important information. Patients were also able to point out the shortcomings of the scale in the pre-survey. For the formal survey, a larger sample (400 patients with GC and 130 healthy subjects) responded to a questionnaire with a reduced number of items (46 items) to improve the rationality of the GC-PROM.

In the development stage of the GC-PROM, we used healthy subjects as a control group to evaluate discriminant validity. The scores of the healthy subjects on the 13 subdomains could be used as baseline values. In the practical application of the GC-PROM, we will evaluate the instrument’s discriminant validity using patients with gastrointestinal diseases and non-GC patients as controls in the future. Concurrent validity was not evaluated as part of the validation stage of the GC-PROM because the simultaneous use of other previous scales in the actual investigation phase may result in estimation bias. And conducting multiple questionnaires will cause some burden to patients with GC, which may increase patient’s boredom and survey cost. Therefore, this study also did not include specific comparison results between this scale and other conventional questionnaires such as EORTC QLQ-STO52 or FACT-Ga. We could not compare the validity between the newly developed questionnaire (GC-PROM) and conventional ones. In the subsequent questionnaire survey, multiple scales of gastric cancer (e.g., GC-PROM, EORTC QLQ-STO52, and FACT-Ga) will be used to evaluate the QoL of patients with GC and compare the concurrent validity. We used a distribution-based method to determine the value of the MCID. In the formal investigation, the repeated-measures sample size was relatively small. These conditions were not very suitable for using the anchor-based method. In future studies, we will further standardize the sample size and the time interval for repeated measurements. Shanxi is a Mandarin-speaking province in northern China. Therefore, in the actual survey, the GC-PROM was in Mandarin, which is the standardized language commonly used in China. This approach ensured that the scale could be used in most areas of China, where Mandarin is used. However, in a few areas of southern China, such as Guangdong and Shenzhen, the most common language is Cantonese. For use in these areas, the newly developed GC-PROM would require further adjustment and verification.

## Conclusions

This project essentially completed the development and validation of the GC-PROM according to the PRO production process stipulated by the United States Food and Drug Administration. GC-PROM can be considered an effective clinical evaluation instrument for patients with GC.

## Supplementary information

**Additional file 1.** Final version of GC-PROM. After two item-selection process based on classical test theory and item response theory, the final GC-PROM consisted of 38 items. It described which items were included in the final scale.

## Data Availability

Please contact the corresponding author for the study data, which will be granted upon reasonable request.

## Abbreviations

CTT: Classical test theory
EORTC QLQ-C30: European Organization for Research and Treatment of Cancer quality of life questionnaire-core questionnaire
EORTC QLQ-STO52: European Organization for Research and Treatment of Cancer quality of life questionnaire-stomach module
FACT-Ga: Functional Assessment of Cancer Therapy-gastric
GC: Gastric cancer
GC-PROM: patient-reported outcomes measure for patients with gastric cancer
IRT: Item response theory
MCID: Minimal clinically important difference
PRO(s): patient-reported outcome(s)
QLICP-ST: quality of life instruments for cancer patients-stomach cancer
QoL: quality of life
RCI: Reliable change index
SEM: Standard error of measurement
