# Supplementary material for: GC-PROM: validation of a patient-reported outcomes measure for Chinese patients with gastric cancer
Source: BMC Cancer. 2020 Jan 16;20:41. doi: 10.1186/s12885-020-6518-z (PMC6966844; doi:10.1186/s12885-020-6518-z)
Supplement: Supplementary file 1 — Additional file 1. Final version of GC-PROM. After two item-selection process based on classical test theory and item response theory, the final GC-PROM consisted of 38 items. It described which items were included in the final scale. [file 12885_2020_6518_MOESM1_ESM.docx]

Additional file 1 Final version of GC-PROM

|  | Never | Occasionally | About half  Of the time | Often | Almost everyday |
| --- | --- | --- | --- | --- | --- |
| A. Physical domain |  |  |  |  |  |
| 1. I have upper abdominal pain. | 0 | 1 | 2 | 3 | 4 |
| 2. I feel worse abdominal pain after eating. | 0 | 1 | 2 | 3 | 4 |
| 3. I will soon feel satiety when I start eating. | 0 | 1 | 2 | 3 | 4 |
| 4. I burp. | 0 | 1 | 2 | 3 | 4 |
| 5. I have a difficulty in eating. | 0 | 1 | 2 | 3 | 4 |
| 6. I have chest pain. | 0 | 1 | 2 | 3 | 4 |
| 7. I have cough and spit. | 0 | 1 | 2 | 3 | 4 |
| 8. I feel fatigue. | 0 | 1 | 2 | 3 | 4 |
| 9. I have loss of appetite. | 0 | 1 | 2 | 3 | 4 |
| 10. I have loss of weight. | 0 | 1 | 2 | 3 | 4 |
|  | **Unable** | **Occasionally** | **About half**  **Of the time** | **Often** | **Almost everyday** |
| 11. I can engage in some hard physical activities, such as lifting heavy bags. | 0 | 1 | 2 | 3 | 4 |
| 12. I can engage in general household duties, such as sweeping and washing clothes. | 0 | 1 | 2 | 3 | 4 |
|  | **Never** | **Occasionally** | **About half**  **Of the time** | **Often** | **Almost everyday** |
| **B、Psychological domain** |  |  |  |  |  |
| 1. I am afraid that my health will be get worse. | 0 | 1 | 2 | 3 | 4 |
| 2. I think the illness is a burden of my family. | 0 | 1 | 2 | 3 | 4 |
| 3. I feel scared without reason. | 0 | 1 | 2 | 3 | 4 |
| 4. I do not want to talk with others. | 0 | 1 | 2 | 3 | 4 |
| 5. I don’t want to talk about my illness to other people. | 0 | 1 | 2 | 3 | 4 |
| 6. I get angry more easily than usual. | 0 | 1 | 2 | 3 | 4 |
| 7. I think my life was meaningless. | 0 | 1 | 2 | 3 | 4 |
| 8. I have no confidence in defeating my disease. | 0 | 1 | 2 | 3 | 4 |
| 9. I lost faith in my future. | 0 | 1 | 2 | 3 | 4 |
|  | **Never** | **Occasionally** | **About half**  **Of the time** | **Often** | **Almost everyday** |
| **C、Social domain** |  |  |  |  |  |
| 1. My family is concerned about my illness. | 0 | 1 | 2 | 3 | 4 |
| 2. My relatives, neighbors and friends all care about my illness. | 0 | 1 | 2 | 3 | 4 |
| 3. My relatives and friends give me material help and support. | 0 | 1 | 2 | 3 | 4 |
| 4. My job is affected because of illness. | 0 | 1 | 2 | 3 | 4 |
| 5. I’m reluctant to take part in the social and family activities because of illness. | 0 | 1 | 2 | 3 | 4 |
| 6. I give up my old hobbies because of illness. | 0 | 1 | 2 | 3 | 4 |
| 7. I care about other bad talks. | 0 | 1 | 2 | 3 | 4 |
|  | **Never** | **Occasionally** | **About half**  **Of the time** | **Often** | **Almost everyday** |
| **D. Treatment domain** |  |  |  |  |  |
| 1.I think my treatment is effective for my illness. | 0 | 1 | 2 | 3 | 4 |
| 2. I have physical improvement after treatment. | 0 | 1 | 2 | 3 | 4 |
| 3. My confidence have increased after treatment. | 0 | 1 | 2 | 3 | 4 |
|  | **Very**  **dissatisfied** | **Dissatisfied** | **Generally** | **Satisfied** | **Very satisfied** |
| 4. I am satisfied with current treatment | 0 | 1 | 2 | 3 | 4 |
|  | **Very**  **unreasonable** | **Unreasonable** | **Generally** | **Reasonable** | **Very**  **reasonable** |
| 5. The medical expenses paid are reasonable. | 0 | 1 | 2 | 3 | 4 |
|  | **Unable** | **Occasionally** | **About half**  **Of the time** | **Often** | **Almost everyday** |
| 6. Follow doctor’s instruction and get rid of bad habits. | 0 | 1 | 2 | 3 | 4 |
| 7. Follow the doctor's advice to take medicines on schedule and at the proper dose. | 0 | 1 | 2 | 3 | 4 |
| 8. I regularly come back to the hospital for a check. | 0 | 1 | 2 | 3 | 4 |
| 9. I can bear the side-effects of the drug. | 0 | 1 | 2 | 3 | 4 |
| 10. I am worried about the side-effects of my medicine. | 0 | 1 | 2 | 3 | 4 |
